# Two separate processes affect the development of the mental number line

**DOI:** 10.3389/fpsyg.2013.00317

**Published:** 2013-06-03

**Authors:** Ronit Goldman, Joseph Tzelgov, Tamar Ben-Shalom, Andrea Berger

**Affiliations:** ^1^Department of Psychology, Zlotowski Center for Neuroscience, Ben-Gurion University of the NegevBeer-Sheva, Israel; ^2^Department of Brain and Cognitive Sciences, Ben-Gurion University of the NegevBeer-Sheva, Israel

Two processes are apparently involved when adults compare magnitudes. One is an analog comparison process, which produces the **distance effect**—a decrease in reaction time (RT) the larger the difference between two compared magnitudes (Moyer and Landauer, 1967). The other is the activation of end stimuli (i.e., objects learned to be representing the smallest or the largest magnitudes in the set), which results in the **end effect**—faster processing of pairs that include the end stimuli of a set (Banks, 1977). Leth-Steensen and Marley (2000) proposed a formal model that shows how the two processes can account for comparisons RTs involving ordinal magnitudes.

Natural numbers are symbolic representations of magnitudes which, at least in adults, are apparently represented along a mental number line (e.g., Dehaene, 1997; Gallistel and Gelman, 2000). Automatic processing allows a direct retrieval of information stored in long-term memory (Logan, 1988; Perruchet and Vinter, 2002) and therefore can be used to examine the mental representation of numbers without contamination by intentionally applied strategies (Kallai and Tzelgov, 2009). This can be seen in Stroop-like phenomena when a task-irrelevant process affects processing of a relevant dimension (Tzelgov, 1997). Automatic processing of numbers can be accessed by a physical size comparison task, in which participants are presented with pairs of numbers differing in the numerical and physical size and instructed to select the physically larger number. The **size congruency effect** (SiCE), referring to faster RT for comparisons of pairs in congruent (e.g., 2_8) compared to incongruent (e.g., 2_8) conditions (e.g., Henik and Tzelgov, 1982), serves as a marker of automaticity of numerical processing. Furthermore, linear increase of the SiCE as a function of intra-pair numerical distance is consistent with an analog representation of numbers (Tzelgov et al., 2013).

Pinhas and Tzelgov (2012) proposed that the two-process model of Leth-Steensen and Marley (2000) also applies to automatic processing of numbers. They attributed the monotonic increase of the SiCE with the intra-pair numerical distance (e.g., Henik and Tzelgov, 1982; Tzelgov et al., 2000) to the analog comparison process. In addition, the faster processing of pairs containing end stimuli was suggested to enlarge the SiCE due to earlier availability of numerical magnitude information (Schwarz and Ischebeck, 2003) and to attenuate the modulation of the effect by the numerical distance. This phenomenon was defined by Pinhas and Tzelgov (2012) as the automatic end effect (AEE), and was assumed to result from real-world experience. The effect shown to exist for 0, and for 1 in the absence of 0, but not for larger numbers; that is, the effect was absent when 2 was the smallest number in the set. This finding is important as it shows the special status of 1 (and 0) as the semantically smallest number stored in long-term memory (Tzelgov et al., 2013) and is consistent with the special status of 1 as hypothesized by Leslie et al. (2008).

While the picture is relatively clear with regard to number representation in adults, less is known about such representation in children. If its emergence reflects learning (e.g., Verguts and Fias, 2008), both processes may be involved in the formation of the mental number line. In particular, we do not know how the processes involved in number comparison (analog number comparison and mapping 1 as the smallest number) develop in children and contribute to the emergence of the mental number line. Thus, the development of these processes is of interest.

Several studies have investigated numerical comparisons in children and used the SiCE to learn about the development of automatization of numerical processing. Rubinsten et al. (2002) reported a numerical distance effect in numerical judgments in kindergarteners (see also Sekuler and Mierkiewicz, 1977) but the SiCE in physical comparisons emerged by the end of first grade, with no modulation by numerical distance. Girelli et al. (2000) classified pairs of numbers as “unilateral” (both numbers smaller or both larger than five) and “bilateral” (one number smaller and the other larger than 5). In numerical comparisons, laterality, being positively correlated with distance, affected latencies in first, third, and fifth graders. In this study, the SiCE was found for third and fifth graders but not for first graders. Zhou et al. (2007) were the only ones to have shown an SiCE modulated by intra-pair numerical distance for Chinese kindergartners, consistent with the assumption of the arrangement of numbers along the mental number line. They attributed the emergence of the effect at this relatively early age to cultural differences.

In the current work we examined the effects of the two comparison processes in both intentional and automatic numerical judgments of children. We used the data of 118 kindergarteners (Mean = 6.1 years, *SD* = 4.2 months) from the study of Ben-Shalom et al. (submitted), where the authors presented children with numerical and physical comparison tasks on stimuli differing in numerical values and physical sizes. Number pairs were presented with intra-pair numerical distance of 1 (the pairs: 1_2, 3_4, 6_7, 8_9), 2 (the pairs: 1_3, 2_4, 6_8, 7_9), and 5 (the pairs: 1_6, 2_7, 3_8, 4_9). Each number in a pair appeared an equal number of times on each side of the screen center. In both tasks, 24 congruent pairs (e.g., 3_8), and 24 incongruent pairs (e.g., 3_8) were created by presenting numbers in physical sizes of 10 mm (small size) or 13 mm (large size). To demonstrate the two processes in both tasks, we conducted separate analyses for pairs that contain 1, and for pairs that did not contain 1. In all analyses the nominal significance level was defined as *p* < 0.05.

In the numerical comparison task (Figure 1A), when pairs that contained 1 and pairs without 1 were included in a common analysis, the children showed a clear distance effect [*F*_(1, 117)_ = 11.29, *MSE* = 26,282, η^2^_*p*_ = 0.09] (see Ben-Shalom et al., submitted). However, pairs containing 1 were compared faster than comparisons of pairs that did not contain 1 [*F*_(1, 117)_ = 46.67, *MSE* = 70,729, η^2^_*p*_ = 0.28]. Furthermore, comparisons of pairs including 1 showed no sensitivity to numerical distance (*F* < 1). In contrast, comparisons of pairs without 1 were faster with the increase in the numerical distance between the numbers in the pair, as indicated by the linear trend for numerical difference (1, 2, and 5) [*F*_(1, 117)_ = 17.05, *MSE* = 34,489, η^2^_*p*_ = 0.13].

**Figure 1:**
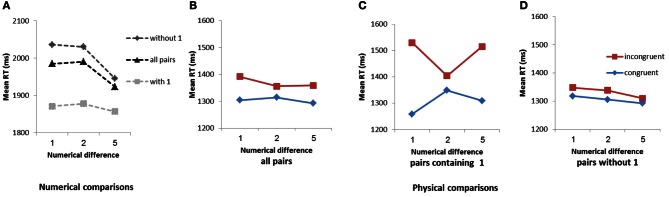
**Mean RTs in the numerical comparison task as a function of pair type and numerical difference (A).** Mean RTs in the physical comparison task as a function of congruency and numerical difference for all pair types **(B)**, for pairs that contain 1 **(C)**, and for pairs without 1 **(D)**.

In the physical comparison task, the SiCE computed for pairs with and without 1 (Figure 1B) was found to be significant [*F*_(1, 117)_ = 28.52, *MSE* = 26,253, η^2^_*p*_ = 0.20] and was not modulated by numerical distance (*F* < 1). In line with the notion of the AAE, the size congruity effect in the physical comparison task was much larger in pairs that contained 1 than in pairs without 1 [*F*_(1, 115)_ = 19.23, *MSE* = 104,335, η^2^_*p*_ = 0.14] (compare **C** and **D** in Figure 1). In fact, the SiCE was apparent only for pairs containing 1 [*F*_(1, 115)_ = 34.06, *MSE* = 166,254, η^2^_*p*_ = 0.23], with no evidence of linear modulation (Figure 1C), and was minimal and marginally significant for pairs without 1 [*F*_(1, 117)_ = 3.85, *MSE* = 32,329, η^2^_*p*_ = 0.03] (Figure 1D). The SiCE for pairs containing 1 did not differ for distances 1 and 5 (*F* < 1) and was larger for the distances of 1 and 5 than for the distance of 2 [*F*_(1, 115)_ = 10.77, *MSE* = 111,129, η^2^_*p*_ = 0.09]. Importantly, an analysis performed on the largest number in the set found no indication for the automatic processing of 9 as an end stimulus, the SiCE for comparisons of pairs containing 9 was non-significant (*F* < 1).

The present study demonstrates the distance effect and the end effect in kindergarteners, showing that in young children as in adults, an analog comparison process and mapping to end anchors are involved in magnitude comparisons (Leth-Steensen and Marley, 2000). Our results replicate the distance effect in kindergarteners (e.g., Sekuler and Mierkiewicz, 1977; Rubinsten et al., 2002) but only in comparisons that did not contain 1. Consistent with Zhou et al. (2007), we also found an SiCE in kindergarteners. The effect was enlarged for pairs including 1, as found for adults (Pinhas and Tzelgov, 2012), and did not increase with numerical distance. In pairs that did not include 1, the SiCE was minimal and insensitive to the numerical distance. It follows that the AEE for 1 and the modulation of the SiCE by intra-pair numerical distance become automatized during development at different rates. Because automaticity is achieved with experience, the fact that only the processing of 1 as an end stimulus affected automatic numerical comparisons suggests that this process develops earlier than the analog comparison process. The finding that 1, but not 9, showed an AEE further implies that 1 has a special status as the smallest member of the mental number line. As kindergartners acquire real-world experience with numbers larger than 9 (e.g., 10) the absence of the effect for 9 may result from such experience. In that sense, it is similar to the absence of the AEE for 2 when it was the smallest number in the set, as reported by Pinhas and Tzelgov (2012).

Leslie et al. (2008) refer to the special role of 1 in the generation of natural numbers. They proposed that humans are born with (1) the ability of symbolic representation of (at least) the minimal possible magnitude by a numeral equivalent to 1, and (2) the function “next,” which recursively allows adding 1 to each (natural) number, thus enabling to generate the representation of each and every natural number. The special status of the number 1 results from the recursive rule, as it is the only number that can be used to generate a representation for each and every natural number in this manner. Our results showing that kindergartners, like adults, show an AEE for 1 (Pinhas and Tzelgov, 2012), implying they process 1 as the smallest magnitude, is in line with Leslie et al.'s assumptions. Assuming the mental number line is created by a next function of the smallest magnitude 1, the formation of the order relations between magnitudes is supposedly created by learning of adjacent pairs. The first order relation to be learned is that the magnitude 1 is smaller than the magnitude 2. In accordance with this suggestion is our finding that in comparisons of pairs containing 1, an enlarged SiCE was found for the numerical distance of 1, that is, comparisons of the pair 1_2.

The special processing of 1 as the smallest magnitude can account for some of the differences found in studies of numerical processing development. Because comparisons of pairs containing 1 show an increased SiCE, the effect may be manipulated by inclusion or exclusion of 1 in the analysis, as demonstrated in this study. In line with our suggestion, Zhou et al. (2007), who found an SiCE for kindergartners, included the number 1 in their stimuli set (in a third of the experiment trials), whereas studies that did not include 1 in the stimuli set showed the emergence of the SiCE only later at the end of first grade (Rubinsten et al., 2002) or in third grade (Girelli et al., 2000).

The insensitivity of the SiCE to numerical distance implies kindergartners do not have an analog representation of numerals as magnitudes in the long-term memory. This does not necessarily mean that magnitudes are not represented mentally along a mental line, but rather the mapping of symbols to this representation is not automatic at this early age.

The earlier development of mapping to end anchors as compared with the analog comparison process is also evident in studies in which the association between symbols and magnitudes is artificially created and learned (e.g., Riley and Trabasso, 1974; Banks, 1977; Tzelgov et al., 2000). The linear ordering of a set is constructed from the ends inward, as participants first learn to identify the end stimuli of the set, and gradually fill in the order relations of stimuli from the rest of the set (e.g., Riley and Trabasso, 1974).

## Summary

The current study showed intentional and automatic numerical judgments of kindergartners are affected by an analog comparison process and the processing of end stimuli. The number 1 was found to have a special status as the smallest number, as implied by both intentional and AEEs. The distance effect was found in intentional comparisons of numbers but was absent in automatic processing. These results indicate the processing of end stimuli develop earlier than the analog comparison process. Finally, we demonstrated the inclusion of 1 in the stimuli set increases the SiCE and suggested this can account for the emergence or absence of the effect as reported in the literature.
